# Cervical Myelitis Masquerading as Guillain-Barré Syndrome: A Case of Acute Flaccid Tetraplegia With a Sensory Level at the Third Cervical Dermatome

**DOI:** 10.7759/cureus.104530

**Published:** 2026-03-02

**Authors:** Yasser Ouatab, Rida Touab, Si Mohammed Andaloussi, Khalil Mounir, Hicham Balkhi

**Affiliations:** 1 Anesthesia and Intensive Care, Mohammed V Military Instruction Hospital, Rabat, MAR; 2 Anesthesia and Critical Care, Mohamed V Military Teaching Hospital, Rabat, MAR; 3 Anesthesiology and Critical Care, Mohamed V Military Teaching Hospital, Rabat, MAR; 4 Surgical Intensive Care, Mohammed V Military Training Hospital, Rabat, MAR; 5 Anesthesia and Intensive Care, Faculty of Medicine and Pharmacy, Mohamed V Military Teaching Hospital, Mohammed V University, Rabat, MAR

**Keywords:** acute flaccid tetraplegia, cervical myelitis, guillain barre syndrome mimic, longitudinal extensive transverse myelitis, neuromyelitis optica spectrum disorder, plasma exchange therapy, rituximab treatment, sensory level, spinal magnetic resonance imaging

## Abstract

Acute flaccid tetraplegia with areflexia and respiratory failure is frequently attributed to Guillain-Barré syndrome in emergency practice. However, high cervical inflammatory spinal cord lesions may present with a similar clinical picture, leading to potential diagnostic delays and inappropriate initial management. A 26-year-old woman presented with sudden interscapular pain followed within hours by rapidly progressive weakness of all four limbs and acute respiratory distress. Neurological examination revealed generalized areflexia, absence of pyramidal signs, and a clearly defined sensory level at the third cervical dermatome. Cerebrospinal fluid examination was normal. Urgent spinal magnetic resonance imaging demonstrated an intramedullary inflammatory lesion extending from the second to the sixth cervical vertebrae, consistent with longitudinal extensive transverse myelitis. Extensive investigations for infectious, autoimmune, and demyelinating causes were inconclusive, and specific antibody testing was negative, limiting etiological characterization. The patient was treated with high-dose corticosteroids, plasma exchange, and rituximab. Despite partial radiological improvement, the neurological outcome remained unfavorable, with persistent severe motor deficit at follow-up. The presence of a sensory level in a patient with acute flaccid tetraplegia is a critical clinical sign that should prompt immediate spinal imaging. Early recognition of cervical transverse myelitis is essential to initiate timely immunotherapy and may influence neurological prognosis.

## Introduction

Acute inflammatory lesions of the cervical spinal cord may produce motor, sensory, and autonomic dysfunction below the level of the lesion [1-5]. Transverse myelitis is an inflammatory disorder of the spinal cord that may extend over three or more vertebral segments, a condition defined as longitudinal extensive transverse myelitis [6-10].

Acute flaccid paralysis associated with areflexia and respiratory distress is classically suggestive of Guillain-Barré syndrome in emergency and intensive care settings [11]. As Guillain-Barré syndrome represents the most frequent cause of acute neuromuscular respiratory failure, this diagnostic reflex is common and often appropriate in early evaluation. However, this heuristic approach may obscure central causes of acute flaccid paralysis. High cervical spinal cord inflammatory lesions can initially present with flaccidity and areflexia before the appearance of pyramidal signs, closely mimicking peripheral neuropathy [1,2,6]. Failure to promptly recognize a spinal origin may delay magnetic resonance imaging (MRI) and the initiation of appropriate immunotherapy, potentially worsening neurological outcomes.

Given the clinical urgency and the therapeutic implications of this distinction, recognizing subtle differentiating signs, such as a clearly defined sensory level, is crucial. We report a case illustrating this diagnostic pitfall and highlight the importance of early spinal imaging in acute flaccid tetraplegia with respiratory involvement.

## Case presentation

A 26-year-old woman with a history of ankylosing spondylitis treated with non-steroidal anti-inflammatory drugs presented to the emergency department with sudden, intense interscapular pain. Within a few hours, she experienced heaviness of both upper limbs followed by progressive weakness of the lower limbs. The neurological deterioration was extremely rapid, with the patient developing complete quadriplegia and progressive respiratory distress on the same day.

On admission, she was conscious and oriented, with an elevated respiratory rate and an oxygen saturation of 85% on room air. Neurological examination revealed complete flaccid tetraplegia with muscle strength graded 0 out of 5 in all limbs, generalized absence of deep tendon reflexes, absence of the Babinski sign, and complete loss of all sensory modalities below a clearly defined sensory level corresponding to the third cervical dermatome. No cranial nerve involvement was noted. This presentation strongly suggested Guillain-Barré syndrome [11,12].

Electrophysiological studies were not performed prior to imaging because of the extremely rapid neurological deterioration and the need for urgent airway protection. Given the presence of a clearly defined sensory level and the suspicion of a cervical spinal cord process, spinal magnetic resonance imaging was prioritized as it would directly influence immediate management decisions. However, the presence of a precise sensory level prompted consideration of an acute cervical spinal cord process, as recommended in the evaluation of acute myelopathies [3]. Respiratory function deteriorated rapidly, requiring endotracheal intubation and mechanical ventilation.

**Figure 1:**
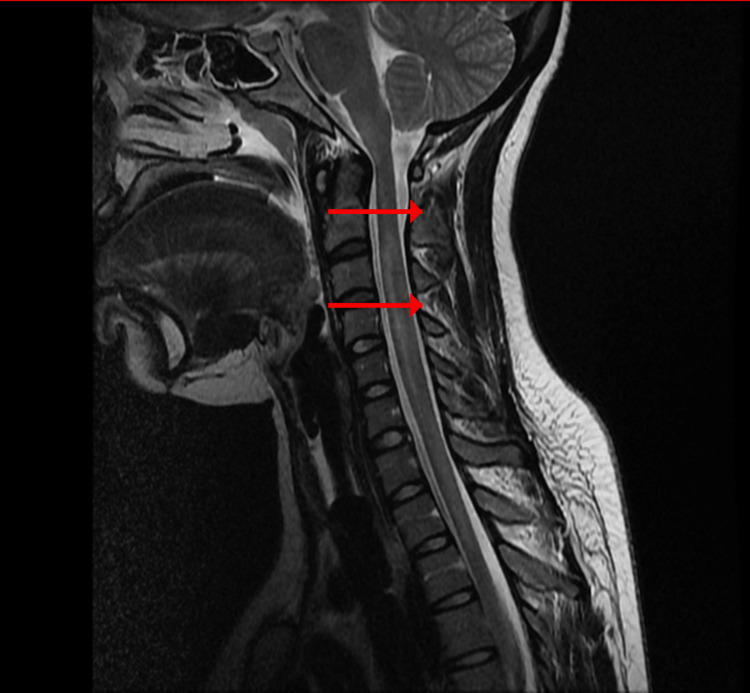
Sagittal T2-weighted cervical spinal MRI showing longitudinal extensive transverse myelitis Sagittal T2-weighted image demonstrating a continuous intramedullary hyperintense lesion extending from C2 to C6 (arrows). MRI: Magnetic resonance imaging.

**Figure 2:**
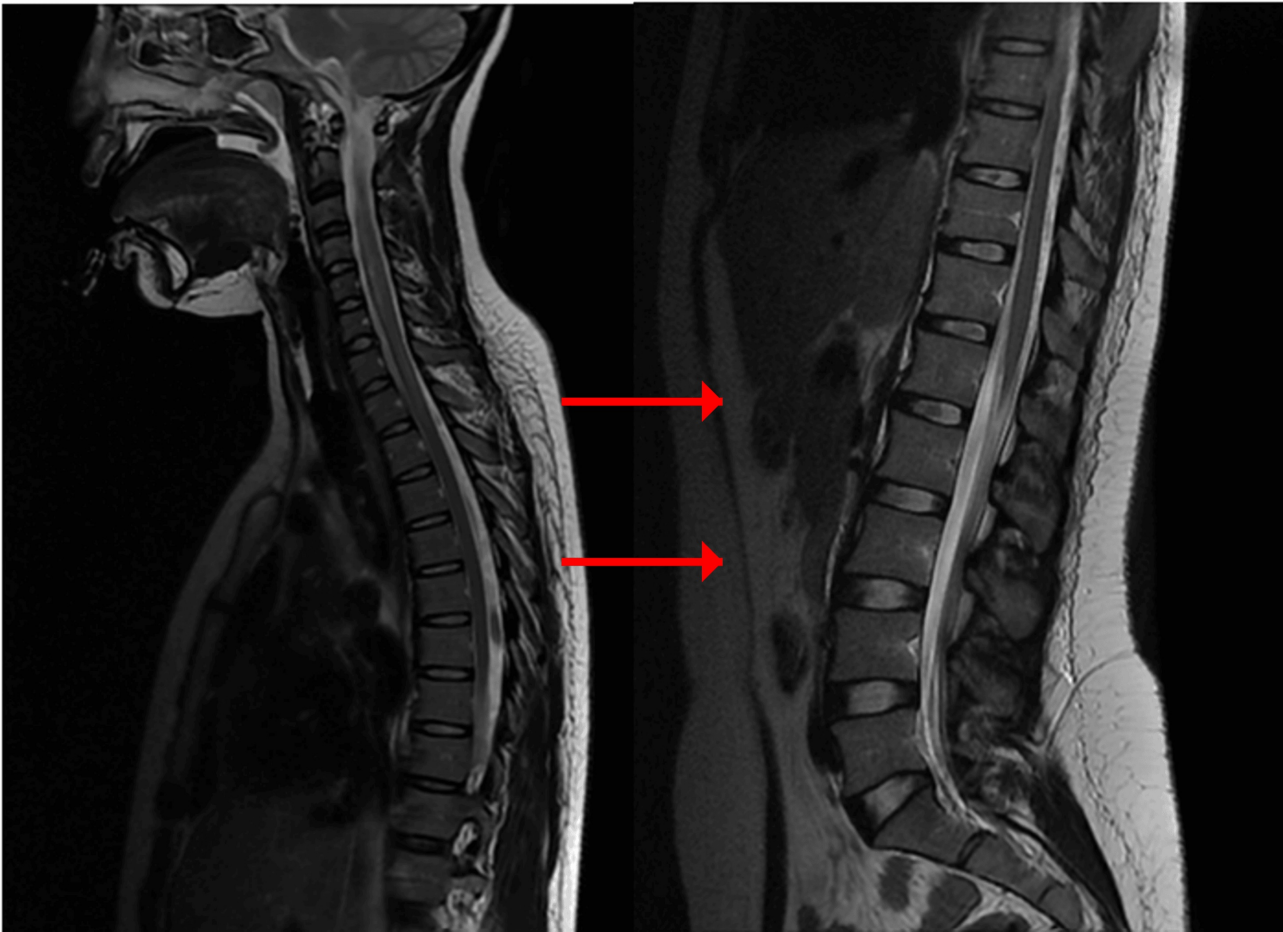
Sagittal STIR cervical spinal MRI confirming inflammatory lesion Sagittal STIR image confirming the inflammatory nature and longitudinal extent of the cervical intramedullary lesion (arrows). STIR: Short tau inversion recovery; MRI: Magnetic resonance imaging

**Figure 3:**
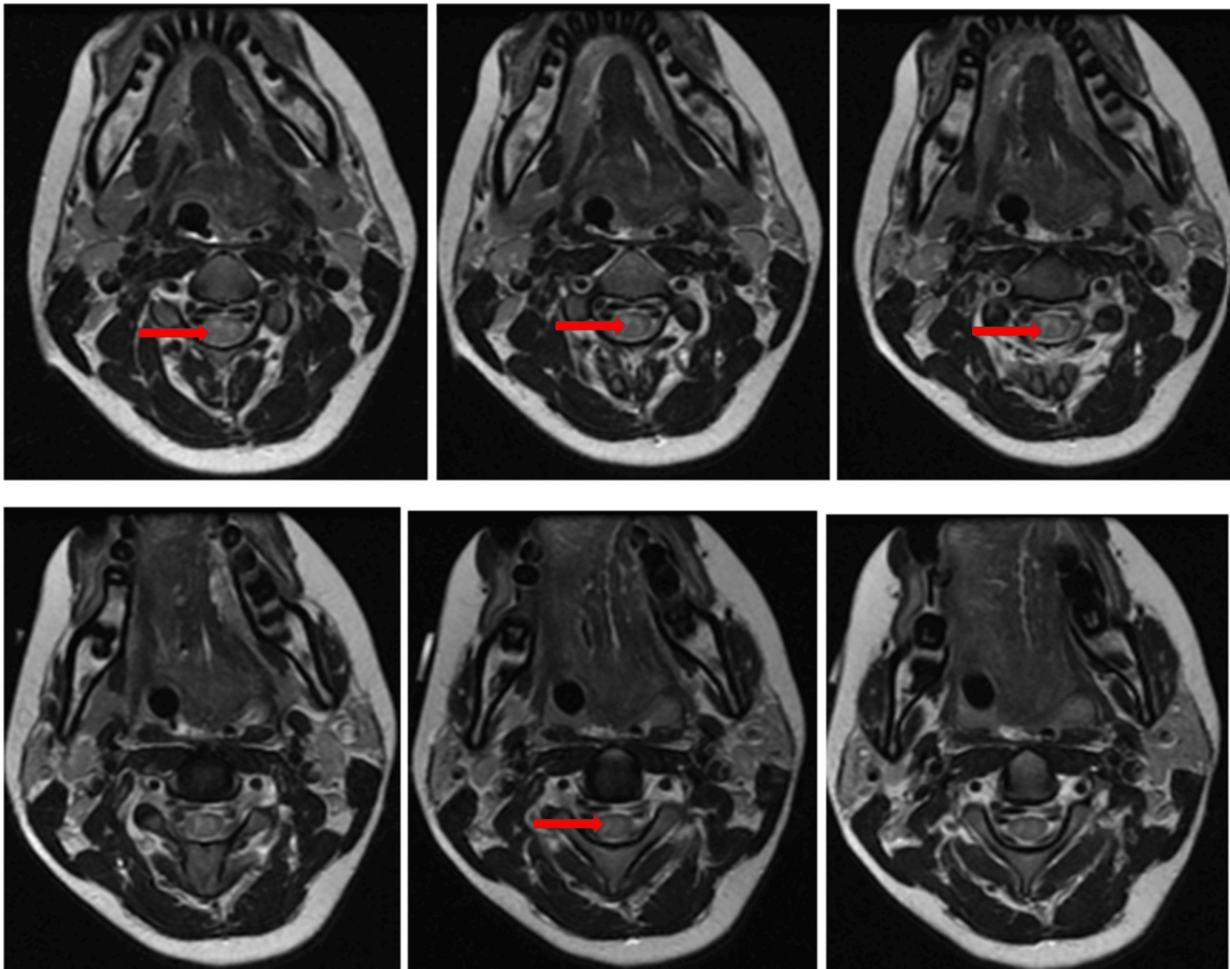
Axial T2-weighted MRI at C3 showing central cord involvement Axial T2-weighted image at the level of C3 demonstrating central intramedullary hyperintensity involving more than two-thirds of the spinal cord cross-sectional area (arrow). MRI: Magnetic resonance imaging

Cerebrospinal fluid analysis showed normal protein concentration and absence of inflammatory cells. This finding argued against typical Guillain-Barré syndrome, where elevated protein concentration without cellular reaction is expected after the first week [12,13]. Urgent spinal magnetic resonance imaging was performed. A sagittal T2-weighted sequence demonstrated a hyperintense intramedullary lesion extending continuously from the second to the sixth cervical vertebrae, consistent with longitudinal extensive transverse myelitis (Figure 1). Short tau inversion recovery sequence confirmed the inflammatory nature and longitudinal extent of the lesion (Figure 2). Axial T2-weighted images at the level of the third cervical vertebra showed central involvement of more than two-thirds of the spinal cord cross-sectional area (Figure 3).

**Figure 4:**
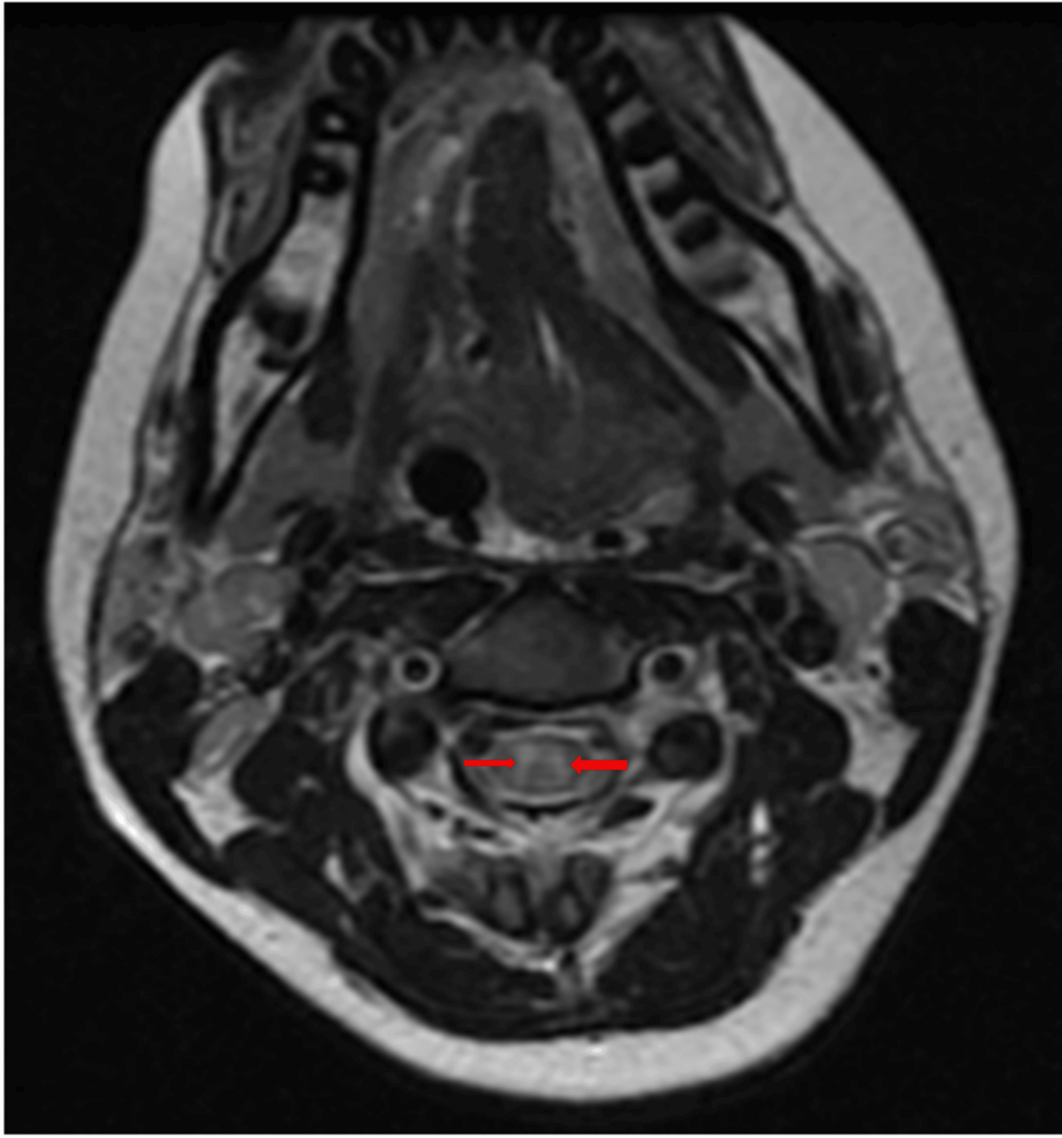
Axial T2-weighted MRI at C5 excluding compressive pathology Axial T2-weighted image at C5 showing diffuse central intramedullary hyperintensity without evidence of extrinsic compression (arrow). MRI: Magnetic resonance imaging

**Figure 5:**
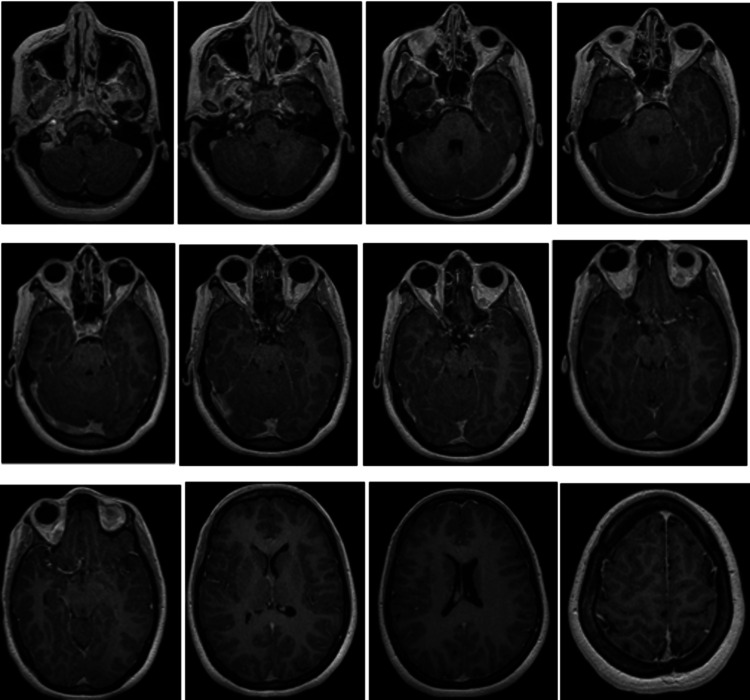
Post-contrast sagittal T1-weighted MRI showing intramedullary enhancement Post-contrast sagittal T1-weighted image demonstrating patchy intramedullary enhancement supporting inflammatory etiology (arrow). MRI: Magnetic resonance imaging

Axial images at the fifth cervical level excluded any compressive pathology (Figure 4). Post-contrast T1-weighted images showed patchy intramedullary enhancement, supporting an inflammatory etiology (Figure 5). Brain imaging was normal.

Subsequently, extensive investigations were conducted. Anti-aquaporin-4 antibodies and anti-myelin oligodendrocyte glycoprotein antibodies were negative. Infectious serologies were negative, while antinuclear antibodies were positive at low titer without a specific autoimmune profile. Thoraco-abdomino-pelvic imaging and salivary gland biopsy were normal.

**Figure 6:**
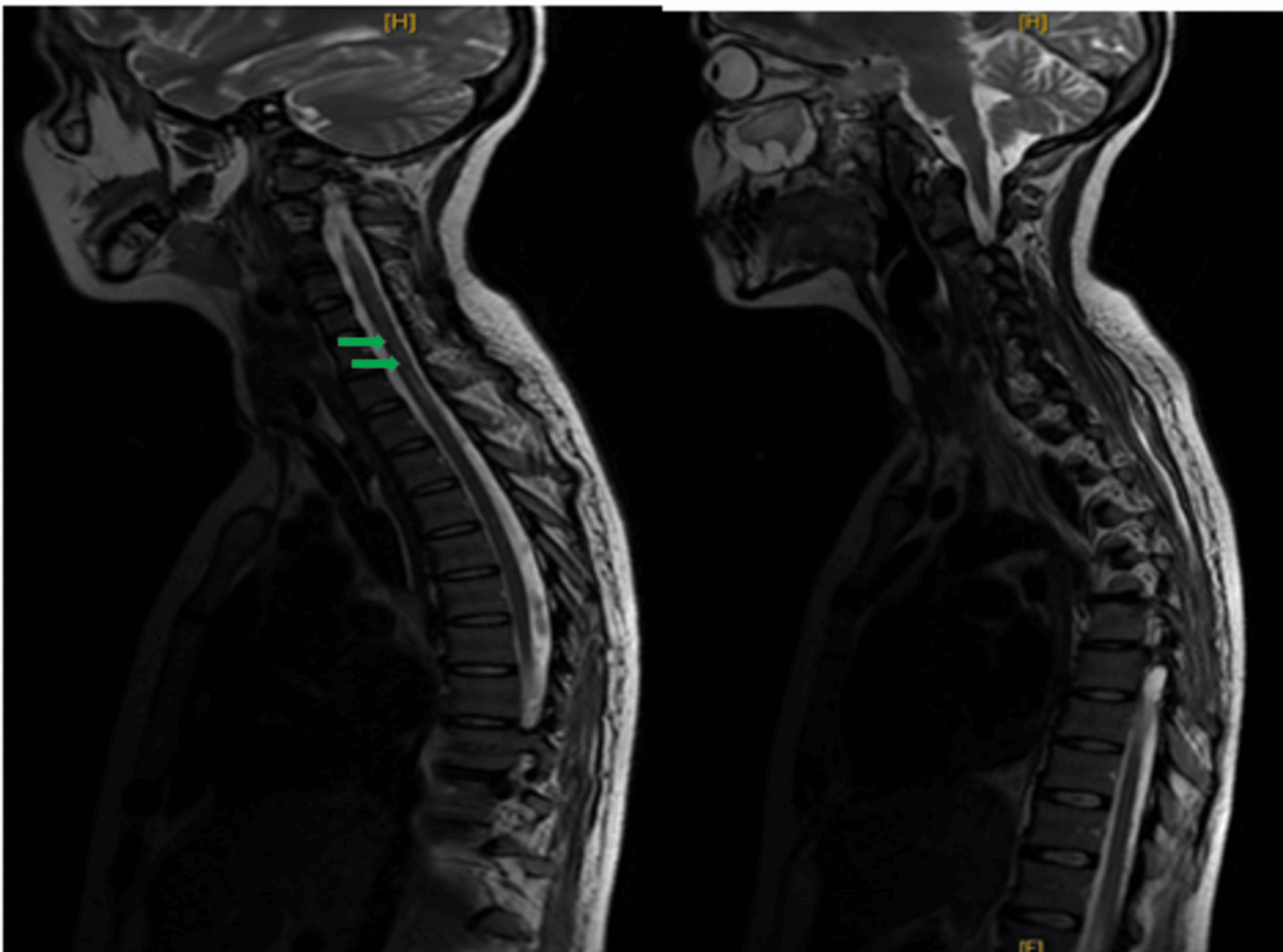
Follow-up sagittal T2-weighted MRI after immunotherapy Follow-up sagittal T2-weighted image showing partial regression of the cervical intramedullary hyperintense lesion after treatment (arrow). MRI: Magnetic resonance imaging

Treatment consisted of intravenous methylprednisolone 1g/day for five days, followed by six sessions of plasma exchange [14,15]. Given the longitudinally extensive nature of the lesion, the severity of the initial presentation requiring mechanical ventilation, and the incomplete early neurological improvement, rituximab was introduced as escalation therapy in the context of suspected severe inflammatory demyelinating disease [16], despite negative antibody testing. Follow-up magnetic resonance imaging after treatment showed partial regression of the lesion (Figure 6).

After prolonged mechanical ventilation complicated by intensive care unit-acquired neuromyopathy, the patient was transferred to neurology. At last follow-up, she had persistent tetraplegia with predominant lower limb spasticity but preserved swallowing and stable general condition.

## Discussion

This case highlights a clinically challenging diagnostic overlap between acute cervical transverse myelitis and Guillain-Barré syndrome [14]. In emergency settings, acute flaccid tetraplegia with areflexia and respiratory failure strongly suggests Guillain-Barré syndrome, as it remains the most frequent cause of acute neuromuscular respiratory failure [11]. However, early spinal cord lesions may transiently present with flaccidity and hyporeflexia due to spinal shock, preceding the development of pyramidal signs [1,2,6].

In our patient, the diagnosis was not based solely on cerebrospinal fluid findings, which may remain normal in early Guillain-Barré syndrome [12,13], but rather on the combination of extremely rapid progression within hours, the presence of a sharply defined sensory level, and the severe cervical pain at onset. This constellation of symptoms was more compatible with an acute cervical myelopathy than with a peripheral demyelinating neuropathy.

Several key clinical elements in this patient argued against a diagnosis of Guillain-Barré syndrome. The extremely rapid progression of neurological deficits within hours is atypical for Guillain-Barré syndrome, which usually progresses over several days [11]. The presence of a clearly defined sensory level is not compatible with peripheral neuropathy and strongly suggests a spinal cord lesion [3,5]. In addition, cerebrospinal fluid analysis remained normal, whereas Guillain-Barré syndrome typically shows elevated protein concentration after the first week without pleocytosis [12,13].

Longitudinal extensive transverse myelitis is defined by a spinal cord lesion extending over at least three vertebral segments. It is strongly associated with inflammatory demyelinating diseases such as neuromyelitis optica spectrum disorders and myelin oligodendrocyte glycoprotein antibody-associated disease [7-9,17]. In contrast, multiple sclerosis typically produces shorter and more peripheral spinal lesions [15,16]. Despite extensive investigations, no precise etiology was identified in this patient. Idiopathic cases of longitudinal extensive transverse myelitis are well documented in the literature [1,10].

The therapeutic implications of this distinction are major. Management of Guillain-Barré syndrome relies primarily on intravenous immunoglobulin or plasma exchange, whereas acute transverse myelitis requires immediate high-dose corticosteroids followed by plasma exchange when necessary [3,5,18,19]. Rituximab has demonstrated efficacy in severe inflammatory spinal cord disorders, and is increasingly used in neuromyelitis optica spectrum disorders and related conditions [20].

In this case, recognition of the sensory level was the decisive clinical element that prompted urgent spinal imaging and avoided further diagnostic delay. Early recognition allowed initiation of appropriate immunotherapy, which may have limited the extent of permanent neurological damage.

Despite early immunotherapy, the neurological outcome remained poor. Several factors may explain this unfavorable evolution, including the extensive longitudinal involvement of the cervical cord, the severity of initial motor deficit, and the delay between symptom onset and initiation of targeted therapy. The need for prolonged mechanical ventilation further contributed to secondary complications, including intensive care unit-acquired neuromyopathy, which may have worsened functional recovery. This highlights that, despite timely recognition and appropriate treatment, longitudinally extensive transverse myelitis may still be associated with a guarded prognosis.

This case emphasizes the importance of a meticulous neurological examination in any patient presenting with acute flaccid paralysis. The identification of a sensory level should prompt systematic evaluation of the spinal cord before attributing the findings to peripheral neuropathy.

## Conclusions

In patients presenting with acute flaccid tetraplegia and respiratory failure, distinguishing peripheral neuropathy from high cervical spinal cord pathology may be clinically challenging, particularly in the early phase before pyramidal signs emerge. The presence of a clearly defined sensory level should raise immediate suspicion of central involvement and prompt urgent spinal magnetic resonance imaging.

This case illustrates how diagnostic uncertainty in the acute setting may significantly influence therapeutic timing and neurological prognosis. Even with early immunotherapy, longitudinal extensive transverse myelitis can result in severe and persistent disability, emphasizing the need for rapid recognition, multidisciplinary management, and realistic prognostic counseling.
